# Visual Recovery Following Linezolid Cessation in an MDR-TB Patient: Detailed Case Analysis

**DOI:** 10.1155/crpu/9939815

**Published:** 2025-07-27

**Authors:** Yovil Bagas Wiyana, Isnin Anang Marhana, Gatot Suhartono, Andreas Haryono

**Affiliations:** ^1^Department of Pulmonology and Respiratory Medicine, Faculty of Medicine Universitas Airlangga-Dr. Soetomo General Academic Hospital, Surabaya, Indonesia; ^2^Department of Ophthalmology, Faculty of Medicine Universitas Airlangga-Dr. Soetomo General Academic Hospital, Surabaya, Indonesia

**Keywords:** linezolid, multidrug-resistant tuberculosis, optic neuritis

## Abstract

Multidrug-resistant tuberculosis (MDR-TB) is characterized by resistance to at least isoniazid and rifampicin. Linezolid is an antibiotic used for drug-resistant Gram-positive bacteria and is a treatment option for MDR-TB. However, its use is associated with optic neuropathy, presenting as acute worsening and bilateral vision loss, typically within 4 months of therapy. A 47-year-old male with MDR-TB relapsed during the sixth month of an individualized treatment regimen at Dr. Soetomo General Academic Hospital, Surabaya. The patient presented with weakness and anemia, receiving a regimen including levofloxacin (750 mg), linezolid (600 mg), clofazimine (100 mg), and cycloserine (500 mg). In the ninth month, the patient developed visual disturbances, initially suspected to be caused by an intracranial tumor. Despite various examinations and treatments, there was no improvement until linezolid was discontinued. The patient's visual complaints gradually improved following the cessation of linezolid therapy. This case underscores the potential for linezolid to cause optic neuropathy during prolonged treatment for MDR-TB. Detailed ophthalmologic examinations, including optical coherence tomography (OCT) and magnetic resonance imaging (MRI), confirmed optic neuropathy without intracranial pathology. Despite high-dose steroid therapy, the patient's vision improved only after 1 month since discontinuing linezolid. This highlights the importance of monitoring for ocular toxicity in patients undergoing long-term linezolid therapy and suggests that timely intervention can prevent permanent visual impairment. The case demonstrates the reversible nature of linezolid-induced optic neuropathy upon drug cessation and emphasizes the need for regular ophthalmologic assessments in patients receiving prolonged linezolid treatment. This report contributes to the understanding of the adverse effects of linezolid and underscores the importance of vigilant monitoring and alternative therapeutic strategies for MDR-TB.

## 1. Introduction

Tuberculosis (TB) is a major infectious disease caused by *Mycobacterium tuberculosis* [1]. It poses a significant global health threat, with cases reported in every country and across all age groups. In 2019, there were 7.1 million reported cases of TB, which decreased to 5.8 million in 2020 due to global efforts to control the disease. Despite these efforts, TB remains one of the leading causes of death worldwide, with approximately 1.5 million TB-related deaths reported in 2020. The emergence of drug-resistant TB, particularly MDR-TB, has added a new layer of complexity to TB control. MDR-TB is defined as TB that is resistant to at least isoniazid and rifampicin, the two most potent first-line anti-TB drugs. The global burden of MDR-TB is substantial, with 201,997 cases reported in 2019, decreasing by 22% to 132,222 cases in 2020. In 2020, 150,359 patients with MDR-TB were undergoing treatment [2].

One of the agents used for patients with MDR-TB is linezolid [3]. Linezolid is an antibiotic that has shown effectiveness against drug-resistant Gram-positive bacteria and has become a critical component in treating MDR-TB. Approved by the US FDA in 2000, linezolid is used for its bacteriostatic activity against TB, with a minimum inhibitory concentration (MIC) of 0.5 *μ*g/mL against *M. tuberculosis* [4, 5]. However, its long-term use is limited due to severe side effects, including neuropathy. The typical dosage for adults with MDR-TB ranges from 300 to 1200 mg every 24 h, but its safety profile diminishes with extended use [3, 6].

This case report focuses on the incidence of optic neuritis in a patient with MDR-TB treated with linezolid. Optic neuritis is a rare (6%–13.2%) but serious side effect associated with linezolid, characterized by acute worsening and bilateral vision loss with variable fundus appearance. This report is aimed at highlighting the clinical presentation, diagnosis, and management of linezolid-induced optic neuritis, providing valuable insights into the risks associated with long-term linezolid therapy. The primary objective of this case report is to document the occurrence of optic neuritis in a 47-year-old male patient with MDR-TB undergoing linezolid treatment. The report will discuss the clinical manifestations, diagnostic procedures, and therapeutic interventions, emphasizing the importance of monitoring for ocular toxicity in patients receiving prolonged linezolid therapy.

## 2. Case Presentation

A 47-year-old male presented to Dr. Soetomo General Academic Hospital in Surabaya with a chief complaint of persistent weakness for 1 week before admission. The patient reported nausea and vomiting following oral antituberculosis drug (OAT) intake, a 4-month history of weight loss, and a 2-week history of decreased appetite. Notably, the patient did not initially exhibit any visual impairments. His TB treatment history includes a 6-month OAT regimen in 2006, deemed a treatment failure, followed by an 8-month treatment in 2007, which was completed successfully at a community health center.

**Figure 1:**
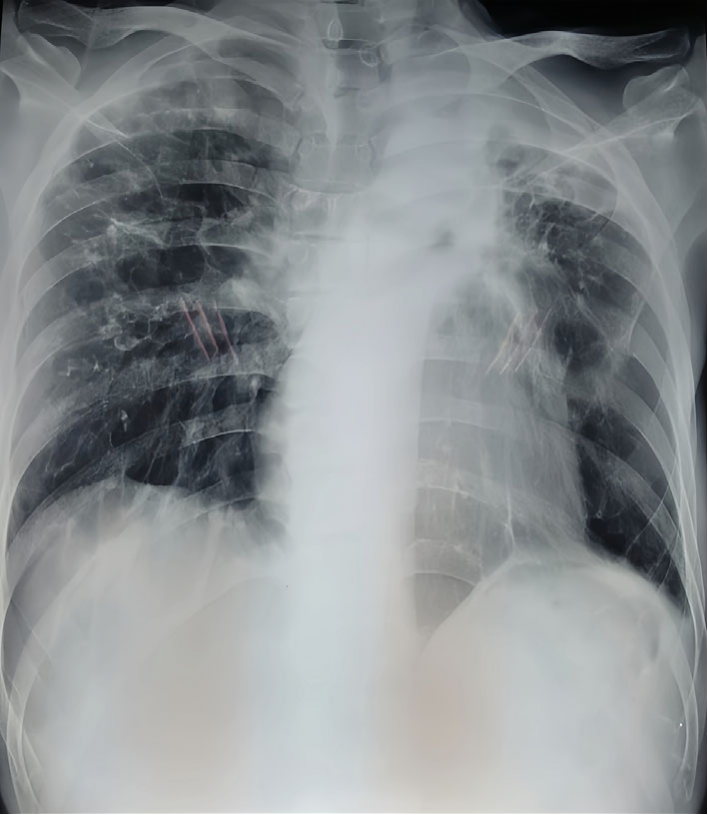
A chest X-ray.

In March 2021, a GeneXpert sputum examination detected high levels of *M. tuberculosis* with rifampicin resistance. Subsequent line probe assay (LPA) indicated sensitivity to fluoroquinolones, while drug susceptibility testing (DST) confirmed resistance to isoniazid (including high-dose isoniazid) and pyrazinamide. Physical examination revealed anemic conjunctiva and abnormalities in the left upper hemithorax, including increased palpable fremitus and dullness on percussion. Chest X-ray (Figure 1) showed tracheal deviation to the left, homogeneous opacity in the upper third of the left hemithorax, rib narrowing, and fibrosis in the left and right lung field. Laboratory tests indicated anemia, leukopenia, and thrombocytopenia.

The patient was diagnosed with weakness, anemia, and MDR-TB on an individualized treatment regimen, along with atelectasis. Nutritional support included a cardiovascular diet of 1900 kcal/day, and treatment involved intravenous fluid therapy (IVFD) with NaCl 1000 mL/24 h and PRC transfusion four bags until hemoglobin levels exceeded 10 g/dL. The MDR-TB regimen initiated in April 2021 for 18 months comprised 4–6 bedaquiline (400–200)–levofloxacin (750)–linezolid (600)–clofazimine (100)–cycloserin (500)/14 levoflaxin (750)–linezolid (600)–clofazimine (100)–cycloserin (500)–B6 (100). The patient also underwent chest physiotherapy.

**Figure 2:**
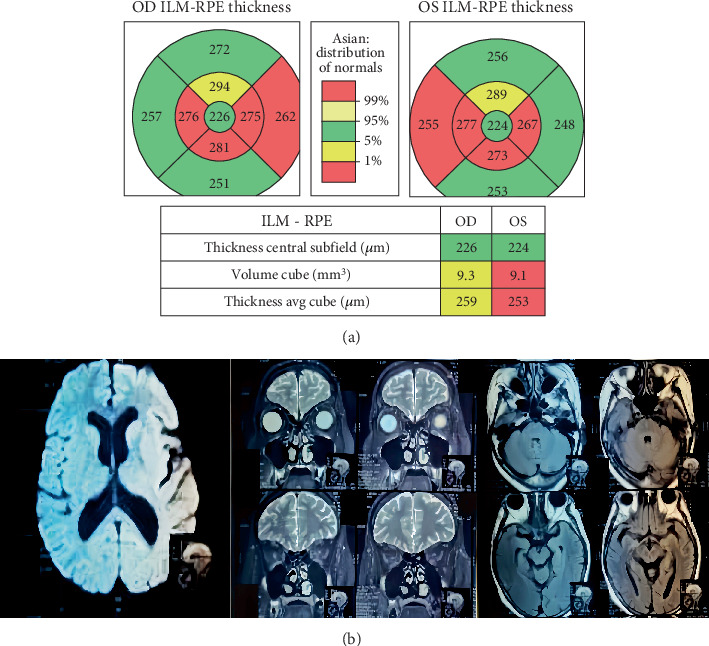
(a) Abnormal macular cube. (b) MRI head focus orbital axial slice.

November 2021, at the 6 months of medication, the patient experienced an improvement in anemia, leukopenia, and thrombocytopenia post-PRC transfusion. However, in the ninth month (February 22), the patient developed visual disturbances. Optical coherence tomography (OCT) revealed optic neuritis (Figure 2a), while MRI angiography showed patent Circulus Willis with no aneurysms, vascular malformations, infarctions, bleeding, or masses in the brain parenchyma (Figure 2b). No abnormalities were detected in the optic nerves on MRI.

Despite high-dose steroid therapy, the patient's vision did not improve. Suspected of having an intracranial tumor by an ophthalmologist, the patient underwent extensive examination and treatment, but without resolution. In the development of the patient during a follow-up at the MDR Clinic of Dr. Soetomo General Hospital on February 7, 2022, it was indicated that there was blurred vision (based on the OCT Macular Cube examination data on February 9, 2022, the result showed optic neuritis).

**Figure 3:**
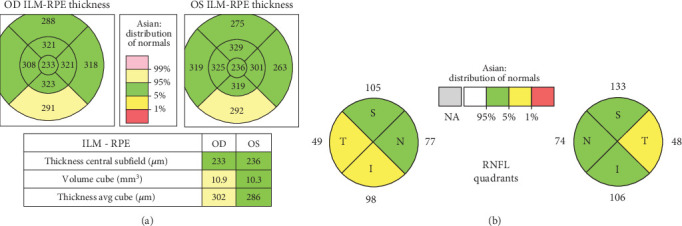
Examination in July 2022 (6 months after stopped the linezolid) showed improvement. (a) Optical coherence tomography macular cube. (b) Optic disc.

Then, 9 months after treatment started, the linezolid regimen was discontinued, leading to gradual visual improvement after 1 month evaluation since discontinuing linezolid.  Figure 3 shows improvement in the paracentral and medial parts of the ocular dextra sinistra (ODS) from the previously thickened macula retina in February 2022.  Figure 3 (July 2022) shows improvement in the inferior part of the optic disc, which previously experienced optic nerve edema in the 8th month of treatment.

In April, the patient began delamanid treatment, which continued for 18 months.

Delamanid was chosen to replace the missing linezolid regimen according to the TB treatment algorithm, as ethambutol is contraindicated in the regimen for group C. The reason for changing the regimen using delamanid was that the patient could not get the linezolid regimen in group A and ethambutol in group C to meet the requirements for administering the regimen.

Treatment with a long-term regimen requires a duration of 18–24 months. The duration of MDR-TB treatment using a long-term regimen is determined based on the timing of culture conversion, with an individualized regimen of 3A+2B + (add-on group C if requirements from Ggroups A and B are not fulfilled) (Table 1). In several previous studies by giving the delamanid regimen replacing the linezolid regimen, there was no data showing significant efficacy. In terms of safety level, delamanid has a better safety level than the use of the linezolid regimen [7, 8].

**Figure 4:**
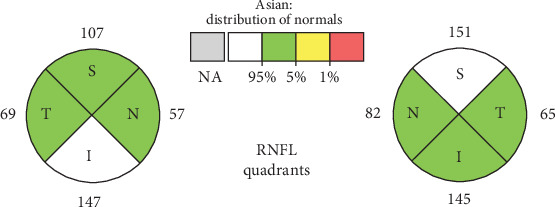
Eye clinic follow-up on February 2022. Blurred vision; the patient underwent an eye evaluation using optical coherence tomography of the optic disc.

The vision condition improved, supported by the results of the eye examination evaluation. The patient was able to return to normal daily activities. The patient also completed an individualized treatment regimen for 18 months, concluding in November 2022 (Figure 4).

## 3. Discussion

Multidrug-resistant tuberculosis (MDR-TB) presents significant challenges in global TB control efforts. Treatment typically involves prolonged regimens with second-line drugs, which are more costly and have severe side effects compared to first-line treatment [7]. The duration of MDR-TB treatment generally ranges from 9 to 24 months, depending on the drug regimen and patient response [8, 9].

Linezolid, a synthetic antibiotic from the oxazolidinone class, has shown efficacy against drug-resistant Gram-positive bacteria and has become an essential component of MDR-TB treatment regimens [3]. Despite its effectiveness, long-term use of linezolid is associated with significant adverse effects, particularly neuropathies [10]. This case report illustrates the development of optic neuropathy in a patient undergoing long-term linezolid therapy for MDR-TB.

The incidence of optic neuropathy among linezolid-treated patients varies, with some studies reporting rates as high as 6%–13.2% [11]. A study in Mumbai, India, found that one in five symptomatic patients was diagnosed with linezolid-associated optic neuropathy, though no statistically significant difference was observed between drug dose and incidence [12]. The variability in fundus appearance and clinical presentation complicates early diagnosis of linezolid-induced optic neuropathy, which occurs during the intensive phase, making the occurrence in this case very rare since it typically happens during the continuation phase [13].

In this case, the patient developed optic neuropathy after 8 months of linezolid therapy, presenting with visual disturbances initially suspected to be due to an intracranial tumor. Detailed ophthalmologic examinations, including OCT and MRI, confirmed optic neuropathy without evidence of intracranial pathology (Figure 2a,b). Despite high-dose steroid therapy, the patient's condition did not improve until linezolid was discontinued, indicating a direct correlation between the drug and the neuropathy [10].

**Figure 5:**
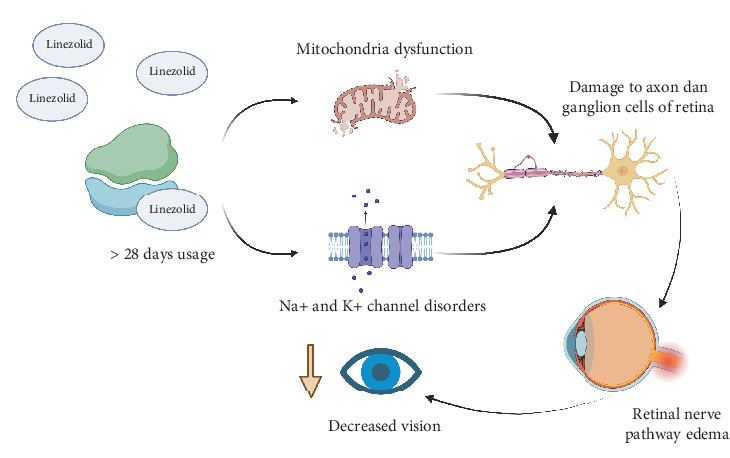
Linezolid pathophysiology of optic neuritis (modified from [10–12, 14]).

The characteristic features of all the reported patients were that they were long-term users of linezolid, and some visual recovery occurred after discontinuing the antibiotic. The mean duration of linezolid treatment was 280 days (range 120–1505 days), which is far from the safety window recommended by the manufacturer (28 days) (Figure 5). However, the use of linezolid is associated with the occurrence of optic neuropathy, with many clinical presentations such as acute, worsening, and bilateral vision loss with variable fundus appearance with incident reports under 4 months. Discontinuation of linezolid treatment resulted in improvement in visual status in all patients. All cellular oxidative metabolism depends on mitochondria. However, the optic nerve may be susceptible to mitochondrial dysfunction. Discontinuation of linezolid treatment resulted in improved visual status in all patients. All cellular oxidative metabolism is dependent on mitochondria. However, the optic nerve may be susceptible to mitochondrial dysfunction [10, 11].

It is evidenced by various categories of optic neuropathies, both genetic and acquired, which have the same clinical picture and result in mitochondrial damage [13–15]. The papillomacular bundle (PMB) consists of parvocellular neurons with some magnocellular neurons. On macular examination, Cube in April showed macular thickening results. Both retinal ganglion cells (RGCs) have a much smaller caliber [13, 14]. Since mitochondria are created and replicated only in the soma, RGCs must transport and distribute these mitochondria in an energy-dependent manner along the optic nerve, all the way to the synaptic terminals in a short time (7–10 days) [15].

The pathophysiology of linezolid-induced optic neuropathy is believed to involve mitochondrial dysfunction. Linezolid inhibits bacterial protein synthesis by binding to the 23S rRNA of the 50S ribosomal subunit, a component absent in mammalian cells, thus exerting minimal direct effects on mammalian protein synthesis [14]. However, long-term linezolid use can disrupt mitochondrial ribosomes, impairing mitochondrial protein synthesis and leading to cellular oxidative stress and dysfunction [15]. This mitochondrial impairment is particularly detrimental to the optic nerve, which relies heavily on mitochondrial energy production for cellular metabolism and function [14, 15]. Disruption of oxidative phosphorylation at any step in the respiratory chain causes significant energy depletion along with the accumulation of reactive oxygen species (ROS) in RGC. This ROS accumulation reduces the electrical potential across the mitochondrial membrane, opening mitochondrial permeability transition pores, which act as apoptosis switches by releasing factors that promote cell death, such as cytochrome c. Mitochondrial dysfunction may also trigger compensating increases in mitochondria, manifesting on OCT as retinal nerve fibre oedema. Oxidative stress usually results from either excessive ROS production, mitochondrial dysfunction, impaired antioxidant system, or a combination of these factors. The prooxidative/antioxidative cellular imbalance between the ROS production and the ability of the biological systems defense mechanisms to eliminate the cellular stress disturbances leads to the vicious circle, since the oxidative stress reciprocally aggravates ROS production. RGC apoptosis after optic nerve injury is caused by lack of neurotrophin support, increased extracellular glutamate levels, disruption of cellular homeostasis, and damage from free radicals. Apoptotic processes are also activated by microglial cells, which release inflammatory mediators (cytokines, prostaglandins, and complement molecules) and ROS [18].

Recent studies have provided further insight into the mitochondrial toxicity of linezolid. Prolonged exposure to linezolid impairs mitochondrial ribosomal function, leading to disrupted protein synthesis and increased production of ROS in RGCs [19]. Mitochondrial dysfunction impacts ATP synthesis, thereby heightening the production of ROS and leading to cellular stress, lipid peroxidation, and ultimately cell death in RGCs [20–22]. These findings suggest that even subclinical mitochondrial impairment may accumulate over time, ultimately leading to optic neuropathy in susceptible individuals.

In addition to the imaging modalities described above, recent literature has emphasized the importance of a comprehensive differential diagnosis in cases of optic neuropathy. MRI characteristics in conditions such as optic neuritis include hyperintensity on T2-weighted images and enhancement patterns following contrast administration [23]. OCT has been utilized effectively in identifying nerve fiber layer thinning associated with neurological diseases, which can easily be misinterpreted as intracranial pathologies if not adequately assessed [24]. These advances underscore the critical role of multimodal imaging in ensuring accurate diagnosis and guiding timely treatment decisions.

This case underscores the importance of monitoring for ocular toxicity in patients receiving long-term linezolid therapy. The potential for reversible visual impairment upon discontinuation of the drug highlights the need for vigilance and timely intervention [11]. Clinicians should consider alternative treatments or dosage adjustments in patients exhibiting early signs of neuropathy to prevent irreversible damage [12].

## 4. Conclusion

This case report details a 47-year-old male patient with MDR-TB who experienced optic neuritis as a significant adverse effect of long-term linezolid therapy. The patient, who had a history of TB treatment failures and resistance, presented with visual disturbances 8 months into his linezolid regimen. Despite high-dose steroid therapy, his vision did not improve until linezolid was discontinued. The patient's visual condition gradually improved after the cessation of linezolid and the initiation of delamanid, which continued until the treatment completion in November 2022.

The findings support previous studies indicating the potential for linezolid-induced optic neuropathy and highlight the necessity for regular ophthalmologic assessments in patients undergoing prolonged linezolid treatment [11, 22]. The case adds to the growing body of evidence on the adverse effects associated with linezolid, reinforcing the need for alternative therapeutic strategies or careful dosage adjustments in long-term use [4, 10].

This case underscores the critical need for vigilance when administering long-term linezolid therapy, particularly in MDR-TB patients. Linezolid, although effective in treating resistant strains of *M. tuberculosis*, poses a risk of severe side effects, including optic neuropathy. The novelty of this case lies in the comprehensive documentation of optic neuropathy as a reversible condition upon the cessation of linezolid. It emphasizes the importance of early detection and intervention to prevent irreversible visual impairment.

Future research should focus on identifying the optimal duration and dosage of linezolid therapy that minimizes adverse effects while maintaining its efficacy against MDR-TB. Studies exploring the mechanisms underlying linezolid-induced optic neuropathy could lead to the development of preventive measures or adjunct therapies to mitigate these risks. Additionally, research into alternative MDR-TB treatment regimens with lower toxicity profiles would be beneficial for improving patient outcomes and quality of life.

## Figures and Tables

**Table 1:** Therapeutic regimen for patients with MDR-TB.

Group A	Levofloxacin/moxifloxacin
	Bedaquiline
	Linezolid

Group B	Clofazimine
	Sikloserin or
	Terizidone

Group C	Etambutol
	Delamanid
	Pirazinamid
	Imipenen-silastatin
	Meropenem
	Amikasin or
	Streptomycin
	Ettionamid or
	Protionamid
	*p-Aminosalicylic acid*

*Note:* Italicized entry indicates the reserve drug used only when other options are unsuitable.

## Data Availability

The data that support the findings of this study are available from the corresponding author upon reasonable request. The data are not publicly available due to privacy or ethical restrictions.
